# A Parallel Multiobjective PSO Weighted Average Clustering Algorithm Based on Apache Spark

**DOI:** 10.3390/e25020259

**Published:** 2023-01-31

**Authors:** Huidong Ling, Xinmu Zhu, Tao Zhu, Mingxing Nie, Zhenghai Liu, Zhenyu Liu

**Affiliations:** School of Computer Science, University of South China, Hengyang 421200, China

**Keywords:** multiobjective clustering, Apache Spark, multiobjective particle swarm optimization (MOPSO)

## Abstract

Multiobjective clustering algorithm using particle swarm optimization has been applied successfully in some applications. However, existing algorithms are implemented on a single machine and cannot be directly parallelized on a cluster, which makes it difficult for existing algorithms to handle large-scale data. With the development of distributed parallel computing framework, data parallelism was proposed. However, the increase in parallelism will lead to the problem of unbalanced data distribution affecting the clustering effect. In this paper, we propose a parallel multiobjective PSO weighted average clustering algorithm based on apache Spark (Spark-MOPSO-Avg). First, the entire data set is divided into multiple partitions and cached in memory using the distributed parallel and memory-based computing of Apache Spark. The local fitness value of the particle is calculated in parallel according to the data in the partition. After the calculation is completed, only particle information is transmitted, and there is no need to transmit a large number of data objects between each node, reducing the communication of data in the network and thus effectively reducing the algorithm’s running time. Second, a weighted average calculation of the local fitness values is performed to improve the problem of unbalanced data distribution affecting the results. Experimental results show that the Spark-MOPSO-Avg algorithm achieves lower information loss under data parallelism, losing about 1% to 9% accuracy, but can effectively reduce the algorithm time overhead. It shows good execution efficiency and parallel computing capability under the Spark distributed cluster.

## 1. Introduction

The clustering algorithm is an unsupervised learning process with many applications in academia and industry. Among the various clustering techniques [1,2], partitioned clustering [3] is widely used because of its low computational requirements. The partition clustering process can be viewed as an optimization problem where the goal is to find the optimal clustering center. McDowell uses a single objective function to evaluate the quality of clustering. Most of these clustering methods use a single objective function to evaluate the clustering results [4]. However, in some real-world problems, it is often necessary to optimize multiple objective functions at the same time [5]. In order to improve the quality of clustering, clustering methods use multiple objective functions to evaluate the generated clusters [6]. This converts the clustering problem into a multiobjective problem (MOP). Research has shown that many multiobjective optimization algorithms have been used in the field of clustering [7,8,9]. Gong proposed an improved multiobjective clustering particle swarm optimization framework [10]. Abubaker proposed a new automatic clustering algorithm based on multiobjective PSO and simulated annealing (MOPSOSA) [11]. Armano and Farmani proposed a multiobjective clustering PSO that can automatically discover the optimal number of clusters [12].

However, with the explosive growth of data, many emerging optimization problems involve handling this data and reducing run time. Traditional data clustering algorithms based on a single machine serial model require scanning and iterative computation of the entire training set, and this process consumes a large amount of computation time and, therefore, cannot be directly applied to large-scale data [13,14]. Yasin Ortakci proposed a multithreaded parallel particle swarm algorithm and applied it to the clustering problem [15]. However, this approach suffers from the problem that when the amount of data is too large, a single machine may not be able to handle it. Hadoop and Spark are some of the new distributed computing frameworks for dealing with big data, and many scholars have successfully applied them to particle swarm clustering problems. Aljarah proposes a clustering method based on parallel particle swarm optimization based on the MapReduce programming model [16]. However, MapReduce requires frequent reads and writes to the hard disk, which makes it unsuitable for iterative processes. On the other hand, Spark is the preferred method for parallel stochastic search optimization algorithms due to its advantages, such as memory-based computing, which makes it faster than Hadoop processing [17]. Wang proposed a parallel clustering algorithm on Apache Spark to deal with the text problem [18]. Chen proposed a Spark Parallel Binary Moth Flame Optimization (SPBMFO)-based algorithm and applied it to the feature selection problem [19]. Govindarajanet proposes data clustering in parallel PSO processing learning analytics system on Spark [20]. In parallel optimization based on a spark, most of them are applied to parallel single-objective optimization algorithms, but few are applied to parallel multiobjective optimization algorithms [21]. Although the distributed computing framework can reduce the running time of algorithms, with the increase of parallelization, how to deal with the uneven data distribution of multiobjective clustering algorithms, which affects the clustering effect, needs further analysis and exploration.

In this paper, we propose a multiobjective PSO weighted average clustering algorithm based on apache Spark (Spark-MOPSO-Avg). First, we propose taking advantage of the benefits of Spark to reduce the iterative reads and writes of the data to improve efficiency by using in-memory operations. Then, we improve the efficiency of the data in the serial port of the algorithm by dividing the whole dataset into multiple partitions, and each particle computes the fitness function on each partition in parallel. Then, a weighted average of the fitness values of all partitions is calculated as the final fitness value to solve the problem caused by random partitioning with unbalanced data distribution when the data are in parallel. In the multiobjective clustering process, Spark-MOPSO-Avg uses the overall clustering deviation metric and the clustering connectivity metric as objective functions to help find the optimal clustering solution and uses accuracy to evaluate the final obtained clustering solution. Based on the accuracy metric, the MOPSO algorithm based on single-machine (Single-MOPSO) and the MOPSO algorithm based on particle parallelism on Spark (Spark-MOPSO-Particle) are then compared.

This paper is organized as follows. In Section 2, we present the mathematical description of the multiobjective clustering problem, the multiobjective particle swarm algorithm, and the basic conceptual background of Apache Spark. In Section 3, the Spark-MOPSO-Avg method is introduced in detail. In Section 4, the experimental study of Spark-MOPSO-Avg on different datasets is discussed. Finally, Section 5 concludes with a summary of the algorithm.

## 2. Background

### 2.1. Mathematical Description of the Multiobjective Clustering Problem

In real life, many problems require considering multiobjective simultaneously to obtain better results. There are many effective methods in the literature to evaluate the effectiveness of clustering. However, since clustering mostly possesses multiple conflicting objectives, finding a general and effective criterion to solve all clustering schemes is difficult. In order to better solve clustering problems, many scholars have considered clustering problems with multiobjective formulations instead of using multiple clustering criteria to optimize simultaneously. Thus, a multiobjective clustering problem [22] can be formalized as (1):

(1) $$C^{*}=\underset{c\in \Omega }{\arg\min}f\left(C\right)$$

where $f\left(C\right)={\left[f_{1}C,f_{2}C,\ldots ,f_{m}C\right]}^{T}$ is the target function vector, and $f_{i}:\Omega \to R$ is the *i*th different (single) optimization criterion, $i\in \left\{1,...,m\right\}$.

To obtain clusters with good compactness and separation. In this paper, two conflicting validity metrics are used as objective functions: one on the compactness of clusters and the other on the connectivity of clusters. A balanced solution is generated by two conflicting clustering objectives, both of which need to be minimized to obtain a better clustering result. The first objective function is called the overall clustering deviation metric [23], which calculates the total distance between data object instances to their corresponding clustering centers. The formula is as follows:

(2) $$Dev\left(C\right)=\sum_{C_{k}\in C}\sum_{i\in C_{k}}\delta \left(i,\mu_{k}\right)$$

where *C* is the set of all clusters, *i* denotes a data point, $\mu_{k}$ is the centroid of cluster $C_{k}$, and $\delta \left(.,.\right)$ is the distance function (e.g., Euclidean distance). (2) shows that the more compact objects in the same class indicate more similar objects in the class and better clustering, so the algorithm aims at minimizing this indicator function. This objective function is biased toward spherical clusters.

We use the clustering connectivity metric [24] to measure how often neighboring objects are assigned to the same cluster as the second objective function. The formula is as follows:

(3) $$Conn\left(X\right)=\sum_{i=1}^{n}\sum_{j=1}^{L}a\left(x_{i},nn_{ij}\right)$$

$$\begin{array}{ccc} x_{i,j} & = & \left\{\begin{array}{c} 1/j,if\notin C_{k}:r,s\in C_{k}\\ 0,otherwise \end{array}\right. \end{array}$$

where *n* is the size of the dataset, $nn_{ij}\left(j\right)$ is the jth nearest neighbor of $x_{i}$, *L* is a constant indicating the number of nearest neighbors of a data point. This objective function emphasizes the nearest neighbor relationship of the data objects, and this objective function can detect galaxy clusters of arbitrary shapes. It can be seen from (3) that this metric should be minimized.

### 2.2. Multiobjective Particle Swarm Optimization Algorithm

The particle swarm algorithm [25] is a heuristic optimization algorithm proposed by Kennedy and Eberhart by simulating the migration and flocking behavior of a flock of birds during foraging. In the particle swarm algorithm, each particle has its velocity and position, where the position information of the particle indicates the potential solution to the problem. The particle finds the optimal solution by the evolution of its position. The velocity $V_{i}$ and the position $P_{i}$ of the particle are updated as follows:

(4) $$v_{i}^{d}=w_{t}\cdot v_{i}^{d}+c_{1}\cdot r_{1}\cdot \left(pbest_{i}^{d}-x_{i}^{d}\right)+c_{2}\cdot r_{2}\cdot \left(gbest_{i}^{d}-x_{i}^{d}\right)$$

(5) $$x_{i}^{d}=x_{i}^{d}+v_{i}^{d}$$

where d=1,2,3,...,D is the particle dimension, $x_{i}$ is the position of the ith particle, $v_{i}$ is the velocity of the ith particle, *t* denotes the number of generations, $w_{t}$ is the inertia weight, $c_{1}$ and $c_{2}$ are the local learning factor and the global learning factor, respectively, $r_{1}$ and $r_{2}$ are random numbers between [0, 1], $pbest_{i}$ denotes the best position before particle *i*, and gbest denotes the global best position currently found in the whole population.

The multiobjective particle swarm optimization algorithm was first proposed by Coello [26]. The basic framework of MOPSO is similar to the criterion of velocity and position update rules for particles and uses a set of external archives to maintain nondominated solutions. Gong [10] proposed a multiobjective clustering framework using particle swarm optimization. In this paper, we use the MOPSO-CD algorithm [27] as a multiobjective optimization framework, where CD stands for crowding distance.

#### 2.2.1. Particle Encoding and Initialization Process for Clustering

**Figure 1:**
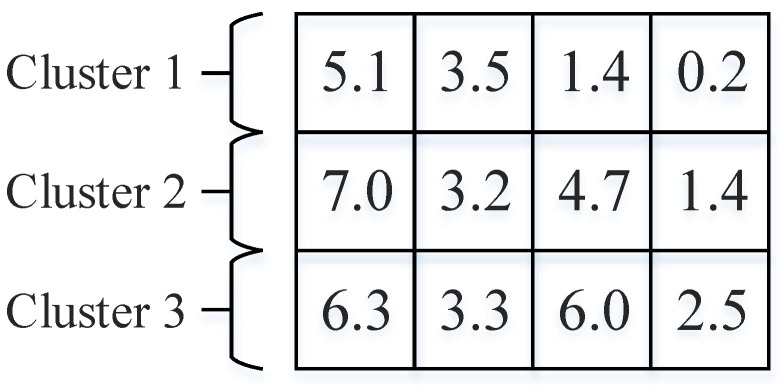
Particle position coding.

In general, particle representation is essential, as each particle represents a solution. In order to solve clustering problems with multiobjective particle swarm algorithms, suitable particle encoding is needed to represent the potential solutions. We use particles as clustering centers so that a single particle encoding representation consists of a two-dimensional matrix of size *K* * *D*, where *K* and *D* denote the number of clusters and the dimensionality of data instances, respectively. For example, a dataset has four attributes and three clusters. Then, the position information of each particle is represented in Figure 1.

A suitable particle swarm initialization mechanism can sufficiently reduce the search space to reach the global best quickly and promote the diversity of solutions. Since the positions in a particle swarm correspond to the cluster centers in a multiobjective clustering problem, the initialization of particle swarm positions may yield undesirable results if they are too random. If the points in the same class are selected as cluster centers simultaneously, this will increase the algorithm time and decrease the algorithm’s efficiency. This paper uses the maximum–minimum distance criterion [28] to initialize the particles. Selecting sample points as close to the clustering center as possible reduces the possibility of using points of the same class as the initial clustering center at the same time.

#### 2.2.2. Crowding Distance

The multiobjective particle swarm base on crowding distance algorithm [29] uses the concept of Pareto dominance to update the external archive and to find the best nondominated solution for the particles. Pareto dominance classifies solutions into dominated and nondominated solutions, and then all nondominated solutions are inserted into the external profile. We also need to update the external archive to ensure the diversity of the data, and after determining the crowding degree values for each particle, we rank the particles according to the crowding distance. The particle with minor crowding is deleted until the external profile is no longer flooded. In addition, to select the global best position, the crowding distance of each particle is first calculated, avoiding the two infinity endpoints, and then the other particles are sorted. The particle with the largest crowding distances is selected as the global best position for subsequent evolution in less crowded regions and thus the diversity of nondominated solutions in the external archive.

### 2.3. Apache Spark

Apache Spark is an open-source distributed computing platform implemented based on the MapReduce programming model. Spark provides an abstract object Resilient Distributed Dataset (RDD) based on in-memory computing. It is known for its scalability, flexibility, and speed [30]. Users only need to read and write their data once and cache it in memory, which can be used repeatedly in later computations. The results of intermediate calculations can be saved in memory, reducing the need for recurrent data reading and writing and drastically improving processing performance. Therefore, Apache Spark has a natural advantage when it is necessary to perform iterative processes, and its performance on some algorithms is more than 10 times that of Hadoop [31].

## 3. Method

A multiobjective particle swarm algorithm is an optimization algorithm in which each iteration relies on the global best solution obtained in the previous iteration as the basis for the evolution of the next particle. As the number of data increases, it becomes impractical for the particles to take longer execution time to compute the fitness values of the objective function in succession. In this section, we propose a parallel multiobjective PSO weighted average clustering algorithm based on apache Spark (Spark-MOPSO-Avg). We reduce the execution time of the algorithm by dividing the data into multiple partitions and computing the fitness values of the particles in parallel. Our goal is to improve computational efficiency while maintaining the clustering quality. In this section, we describe the main components of the proposed method in detail, giving the pseudocode of Spark-MOPSO-Avg.

### 3.1. Fitness Evaluation

The proposed fitness values parallel computation incorporates a weighted average operation on the data, which can also effectively extract information about the local fitness values when the data distribution is unbalanced. The fitness values parallel calculation is divided into two portions in this article. The first part calculates the local fitness values. Since each node has a part of the overall data, the data in each node is only involved in the local fitness values calculation. There is no need to transmit many data objects between nodes in the clustering process, reducing the communication of data in the network. The main idea of the algorithm is: in the master node, the initialized particle swarm will be to broadcast each worker node. Each worker node first uses the position information of the particle as the cluster center and predicts the data in the partition by k-means to get the clustered index corresponding to the data object in that partition at the particle position. Then, the clustering indexes of the data objects of each node are calculated according to (2) and (3) to obtain the local fitness values. This process does not need to transmit a large amount of data, only the data of particle clusters, so the network communication is small, and the execution is efficient.

In the second part, after calculating the local fitness values, we obtain different local fitness values for each particle in each node. Then, we need to aggregate the different local fitness values obtained from each node to the master node by Spark’s collect() function. Since the data quality of each node is different, in this paper, we consider adding up the fitness values of particles *i* for each working node in the master node to obtain the total fitness value of particles $F_{i}$. It can be formalized as (6).

(6) $$F_{i}=\sum_{c=1}^{n}f_{c,i}*\frac{N_{c}}{N}$$

where $f_{\left(c,i\right)}$ denotes the local fitness value obtained by training particle *i* in partition *c*, $N_{c}$ denotes the number of samples in partition *c*, and n denotes the total number of samples in all partitions.

### 3.2. Spark Implementation of Multi-Objective Particle Swarm Algorithm

**Figure 2:**
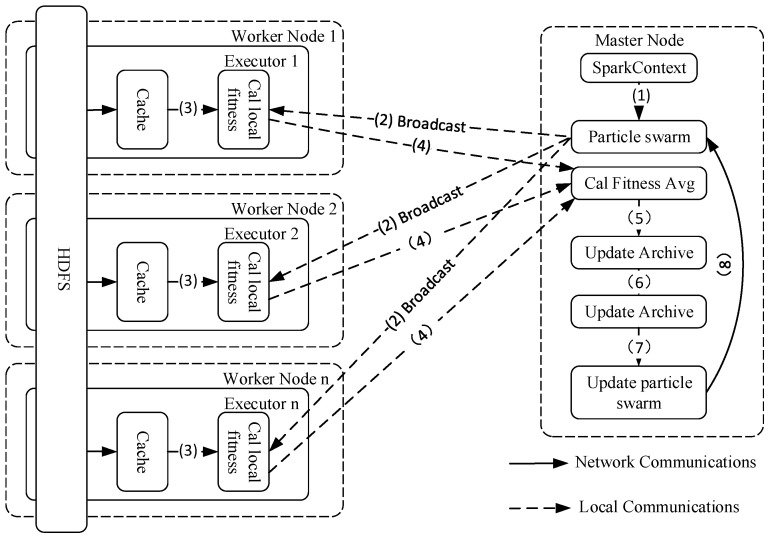
Spark-MOPSO-Avg Architecture.

Figure 2 depicts the general framework for parallel particle swarm-based multiobjective clustering on Spark. First, an initial particle swarm is generated in the master node, and then all particles are sent to each node’s executor via the cluster manager using broadcast variables. Each executor then reads a portion of the HDFS data and caches it in the memory of its respective worker, which is encapsulated in an RDD. Each executor reads a portion of the data from the memory of their respective worker and the particle information obtained from the broadcast to perform the particle fitness calculation. When the computation task of all the executors is completed, the particle fitness values are weighted and aggregated in the master node. Then, operations such as updating the external archive, the optimal solution, and the particle population are performed. Then, the updated particle swarm is rebroadcast to the actuators of each node to start a new iteration. Until the maximum number of iterations is reached, the cycle ends and belongs to the final Pareto solution set.

Algorithm 1 shows the proposed multiobjective particle swarm clustering algorithm based on Apache Spark, which mainly consists of the following steps. A related implementation of this algorithm is available on GitHub https://github.com/HadwinLing/Apache-Spark-MOPSO-Clustering (29 December 2022).

Step 1: Read HDFS data and create RDD. Read the dataset to be trained from HDFS to create dataRDD with *n* partitions, each partition with m/n data instances.

Step 2: Initialize the population. Generate $N_{p}$ initial clustering centers from dataRDD as a particle’s position information. Moreover, randomly generate the velocity information of the particles. Broadcast from the master node to each worker node.

Step 3: Compute the fitness values of the particles in parallel. Broadcast the particle swarm and calculate the fitness values of the particles in parallel using Algorithm 2.

Step 4: Update pbest. Suppose the current particle fitness dominates the current $pbest^{\prime }s$. If it does, replace the $pbest^{\prime }s$ fitness value and position with the particle’s fitness value and position. Otherwise, no change.

Step 5: Update the archive. Copy all the particles in the particle swarm to the archive, then use the Pareto dominance concept to find the best nondominated solution for the particles and insert all the nondominated solutions into the external archive. After calculating the crowding degree value for each particle, the ranking is done based on the crowding distance. The particles crowding the minimum crowding distance are eliminated until the external archive is overflowing.

Step 6: Determine whether the number of iterations reaches the specified number, and if so, output the external archive; if not, proceed with the following evolution:

1. Update the optimal global solution. The choice of the optimal global solution affects the evolutionary direction and diversity of the particles. The optimal solution is computed by first calculating the crowding distance of each particle, then avoiding the two infinity endpoints and ranking them. Those particles with the largest crowding distances are selected as the optimal solutions for subsequent evolution in less crowded regions and thus the diversity of nondominated solutions in the Archive.

2. Update particle swarm. The position and velocity of the updated particles are performed according to (5) and (4).

Step 7: Iteration number +1, return to step 3.

**Algorithm 1** Spark-MOPSO-Avg.
**Input:** dataset, Repository, Archive, Itermax, n, $N_{p}$
**Output:** a group of external archives
    1:// Run in Master Node    2:dataRDD ← sc.textFile(hdfs).repartition(n).persist()    3:particleSwarm ← Initialize particle swarm    4:Archive ← Initialize Archive    5:particleSwarmBC ← sc.broadcast(particleSwarm)    6:// Calculate the fitness value of the particle using Algorithm 2 and $localData_{c}$ is the data cached in the working node c    7:localFitnessRDD ← dataRDD.mapPartition(calFitness($localData_{c},particleSwarmBC$))    8:localFitness ← localFitnessRDD.collect()    9:**for** each particle in particleSwarm **do**  10:    particle’s $Fitness_{i}$←$\sum_{c=1}^{n}localFitness_{c,i}*\frac{N_{c}}{N}$  11:**end for**  12:particleSwarm.map (Update pbest)  13:Archive ← Update Archive  14:**for** each iter = 1, 2, …, Itermax **do**  15:    gbest ← Select Global Best Fitness  16:    **for** each particle in particleSwarm **do**  17:        Update particle velocity by (4)  18:        Update particle position by (5)  19:    **end for**  20:    particleSwarmBC ← sc.broadcast(particleSwarm)  21:  // Calculate the fitness value of the particle using Algorithm 2 and $localData_{c}$ is the data cached in the working node c  22:  localFitnessRDD ←dataRDD.mapPartition(calFitness($localData_{c},particleSwarmBC$))  23:    localFitness ← localFitnessRDD.collect()  24:    **for** each particle in particleSwarm **do**  25:        particle’s $Fitness_{i}$←$\sum_{c=1}^{n}localFitness_{c,i}*\frac{N_{c}}{N}$  26:    **end for**  27:    **for** each particle in particleSwarm **do**  28:        **if** particle’s Fitness Pareto dominates pbest **then**  29:           Update pbest  30:        **end if**  31:    **end for**  32:    particleSwarm.map(Update pbest)  33:    Update Archive  34:**end for**  35:return Archive



**Algorithm 2** calFitness Algorithm.

**Input:** $localData_{c},particleSwarmBC$


**Output:** particleSwarmFitness
    1:// Run in worker Node c    2:**function**calFitness($localData_{c},particleSwarmBC$)    3:    particleSwarmBCValue = particleSwarmBC.value    4:    **for** 1 ≤ *i* ≤ particleSwarmBCValue **do**    5:        dataWithClusterK ← K-means clustering with $particle_{i}$.position as the cluster center using $localData_{c}$    6:        Calculate the objective function value as the local fitness value based on (2) and the dataWithClusterK    7:        Calculate the objective function value as the local fitness value based on (3) and the dataWithClusterK    8:    **end for**    9:    return particleSwarmFitness  10:
**end function**



## 4. Experiment

This section summarizes the methods used to evaluate and compare the experiments and describes the data set, setup, and experimental conditions used during this study.

### 4.1. Experimental Environment

The platform is built and experimentally validated in the Spark framework. The platform consists of one master node and four slave nodes, which all have the same hardware and software configuration. All nodes have Inter(R) Xeon(R) Glod 5215 CPUs with 40-core and 240 GB of RAM on the hardware side. On the software side, each node has 18.04.1 Ubuntu OS, JDK version 1.8.0.131, Hadoop version 2.10.0, Spark version 3.0.0, and Scala version 2.12.10 installed.

### 4.2. Datasets

The experiment has been validated on several real datasets. We have used datasets from the OpenML datasets repository https://www.openml.org/ (29 December 2022). Table 1 summarizes the main features of these datasets, including the number of attributes, the number of instances, and the number of clusters.

### 4.3. Parameter Setting

Table 2 gives the values of the critical parameters of Spark-MOPSO-Avg, which are used to tune the algorithm’s performance. $N_{p}$ parameter indicates the number of particles in the particle swarm algorithm. The Itermax parameter indicates the maximum number of iterations of the algorithm. The Repository parameter indicates the maximum number of nondominated solutions stored in the Archive. *n* represents the number of partitions. *w* parameter indicates the weight factor. $c_{1}$ and $c_{2}$ parameters are the particle learning parameters.

### 4.4. Comparing Clustering Algorithms

On the above dataset, the proposed Spark-MOPSO-Avg algorithm is compared with the MOPSO algorithm base on single-machine (MOPSO-Single), the MOPSO algorithm base on particle parallelism based on Spark (Spark-MOPSO-Particle), and the MOPSO algorithm based on label partitioning on Spark (Spark-MOPSO-labelPartition).

The MOPSO algorithm based on a single machine (MOPSO-Single) is written in Scala, and the optimization functions are consistent with Spark-MOPSO-Avg, while the algorithm runs on a single machine.

The MOPSO algorithm-based particle parallelism on Spark (Spark-MOPSO-Particle) is to parallelize particles and uses the same optimization function. The particles are initialized to generate a particleRDD, and the broadcasted data is then used for iterative computation.

The MOPSO algorithm based on label partitioning on Spark (Spark-MOPSO-labelPartition) is the same as Spark-MOPSO-Avg. Since Spark-MOPSO-Avg has the problem of unbalanced data distribution when partitioning data randomly, Spark-MOPSO-labelPartition partitions the data based on labels only to simulate this situation.

### 4.5. Results and Analysis

To test the effectiveness of the parallelized Spark-MOPSO-Avg algorithm, we perform tests on the Phoneme, Kin8nm, and mozilla4 datasets. Each record of these three datasets has a corresponding label. We compared the accuracy of the proposed Spark-MOPSO-Avg, Spark-MOPSO-Particle, and MOPSO-Single by putting them to the test on the above three datasets. During the experiments, the three datasets are run 10 times, and the average value is taken as the final experimental result data to reduce the effect of random errors.

#### 4.5.1. Experiment 1: Running Time Comparison

The running time metricis used to evaluate whether the improved algorithm improves the effectiveness of the algorithm, and it determines how fast the algorithm runs. In this paper, we use runtime to measure the execution efficiency of the Spark-MOPSO-Avg algorithm.

**Figure 3:**
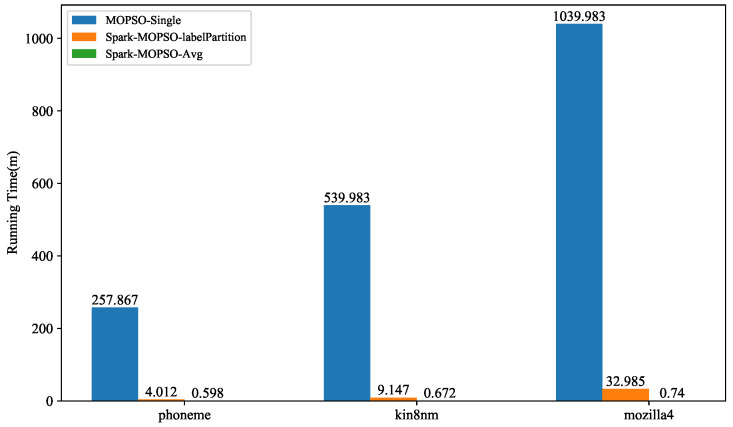
Running Time values for each algorithm processing dataset.

Figure 3 shows the execution time of the above three algorithms under Phoneme, Kin8nm, and mozilla4 datasets. It can be seen that Spark-MOPSO-Particle reduces the clustering time more than MOPSO-Single. However, as the data size increases, the efficiency improvement of Spark-MOPSO-Particle is not significant. In contrast, for Spark-MOPSO-Avg, the computational efficiency is greatly improved over MOPSO-Single and Spark-MOPSO-Particle. Spark-MOPSO-Avg will first partition the data and cache it in memory. Apache Spark broadcasts the particles to each node, and each node reads data from local memory for calculation each time. The algorithm only calculates local data, which reduces the communication overhead of data in each node, so it greatly reduces the clustering time. Spark-MOPSO-Particle reads the dataset from a file and uses Spark’s Broadcast to broadcast the entire dataset to all nodes and cache it in memory. When it encounters an execution operator, it reads the data directly from memory and computes it. However, when the data volume is large, the memory of a node cannot cache all the data, and the algorithm scans the whole data for computation when executing a serial algorithm, which greatly reduces the operational efficiency and even prevents the algorithm from running.

Table 3 shows the results of the statistical analysis of the Wilcoxon test results of the running time metric between the Spark-MOPSO-Avg algorithm and other algorithms. In the Wilcoxon test, ✘ indicates when the *p*-value is greater than 0.05 and the same distribution may exist between the two algorithms; otherwise, ✔ is used to indicate that there may be significant differences between the two algorithms. Based on the Wilcoxon test results of the running time metric, there is a significant difference between the running time of the Spark-MOPSO-Avg algorithm and other algorithms on the three datasets.

#### 4.5.2. Experiment 2: Accuracy Rate Comparison

In order to evaluate clustering results, therefore, an accuracy rate is used to evaluate the clustering result algorithm. This paper uses datasets with labels. The accuracy rate is defined in (7).

(7) $$p=\frac{R}{N}$$

where *R* is the number of data objects with the same labels as the labels of the corresponding clusters, and *N* is the total number of data objects. The accuracy rate can visually evaluate the clustering results of the algorithm. When the value of *P* is larger, it indicates that the clustering results are more accurate and reasonable.

Table 4 gives the accuracy rate of Spark-MOPSO-Avg, Spark-MOPSO-Particle and MOPSO-Single for three different datasets with five nodes. Table 4 shows that MOPSO-Single and Spark-MOPSO-Particle are more accurate than Spark-MOPSO-Avg in handling the clustering problem because both algorithms involve all sample data of the entire dataset in the iterative computation process. For Spark-MOPSO-Avg, only the data in the partitions are involved in each iteration, so the accuracy is not as good as that of MOPSO-Single and Spark-MOPSO-Particle, which use all the data in the computation. However, Spark-MOPSO-Avg obtains lower information loss when dealing with clustering problems than MOPSO-Single and Spark-MOPSO-Particle, with only about 1% to 9% accuracy loss compared to MOPSO-Single. This indicates that Spark-MOPS-Avg achieves low error in processing the clustering problem.

Table 5 gives the accuracy of Spark-MOPSO-Avg and Spark-MOPSO-labelParition. From the table, it can be seen that the accuracy loss of Spark-MOPSO-labelPartition is about 3% to 5% compared to Spark-MOPSO-Avg. This indicates that Spark-MOPSO-avg can also obtain lower loss in the data skewing problem caused by Spark partitioning.

Table 6 shows the Wilcoxon test result for the accuracy rate metric between the Spark-MOPSO-Avg algorithm and other algorithms. For the two datasets, phoneme and Kin8nm, there is the same distribution in accuracy rate metric between the algorithm proposed in this paper and the other algorithms. For the Mozilla4 dataset, although the *p*-value between Spark-MOPSO-Avg and the other algorithms is less than 0.05, the combination of running time metric and accuracy rate metric considerations shows that Spark-MOPSO-Avg significantly reduces the processing time in the algorithm runtime. Therefore, based on the accuracy rate metric, the Spark-MOPSO-Avg algorithm obtains a lower accuracy loss relative to the other algorithms but significantly improves computation time, indicating that the algorithm is capable of accomplishing the task of big data clustering.

#### 4.5.3. Experiment 3: Scalability Comparison

In order to evaluate the scalability of the Spark-MOPSO-Avg algorithm, the speedup ratio is used to evaluate the algorithm. The scalability of Spark-MOPSO-Avg is evaluated by varying the number of nodes in the cluster. This experiment uses different sizes of data object sets for comparative analysis. The experimental data are generated using standard Blobs data generated by Python Sklearn’s make_blobs() function in a square region in the *x*-range [−10, 10] and *y*-range [−10, 10], and the sizes and data volumes of the data object sets are shown in Table 7.

**Figure 4:**
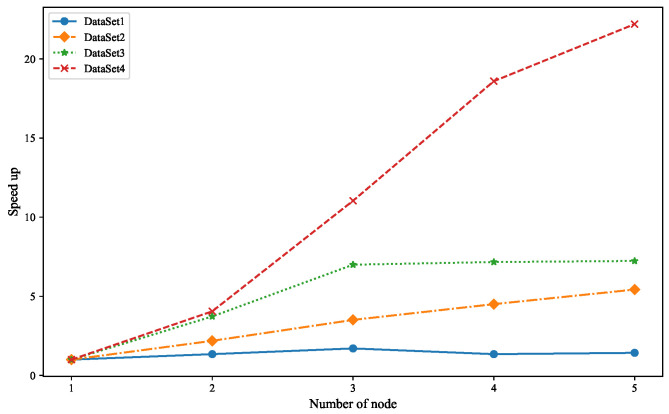
Speedup results of Spark-MOPSO-Avg on simulated data sets.

The Speedup rate metric measures the parallelization capability of an algorithm by calculating the ratio of the running time on a single node to the running time on parallel nodes. The rate of Speedup is determined as follows, where the data set size is constant, and the number of nodes is gradually raised. The Speedup is defined as in (8).

(8) $$Speedup=\frac{T_{1}}{T_{m}}$$

where $T_{1}$ denotes the algorithm’s running time on a single node, $T_{m}$ denotes the running time of parallel computation, and *m* is the number of nodes. A larger Speedup indicates the higher parallelization efficiency of the algorithm. To verify the parallel performance of the Spark-MOPSO-Avg algorithm, four datasets of different magnitudes in Table 7 are used in the experiments. The experiments are conducted by controlling the number of Spark clusters at the number of nodes 1–5, respectively. The experimental results are shown in Figure 4.

From the comparison in Figure 4, we can see that for four different datasets, the Spark-MOPSO-Avg algorithm has varying degrees of parallel running time reduction as the number of nodes increases and the speedup ratio also rises. This means that the algorithm’s parallel speedup effect is also better as the number of Spark cluster nodes rises. Theoretically, each time the number of Spark cluster nodes is doubled, the parallel execution time of the algorithm should be reduced by half. However, as the number of running nodes increases, the network communication overhead between nodes also increases the running time. Thus, the speedup ratio grows slowly with the number of nodes. The larger the dataset is, the more significant the speedup effect is for the same nodes. This experiment can show that the Spark-MOPSO-Avg has good parallel performance when performing operations on large data sets in parallel.

## 5. Conclusions

In big data research, the clustering problem has been an important area. In this paper, we propose a parallel multiobjective PSO weighted average clustering algorithm based on Spark (Spark-MOPSO-Avg). The algorithm uses the multiobjective particle swarm algorithm as the basic framework, employs two conflicting validity metrics as the objective functions, and computes the fitness values in parallel using Spark. The proposed method was evaluated on real and simulated datasets and showed promising results based on running time, accuracy rate, and speedup metrics. It is shown that the multiobjective particle swarm algorithm for data clustering can perform well in a parallel environment. In future work, we propose to use streaming data in multiobjective PSO or other multiobjective population intelligence optimization algorithms for data clustering or apply it to other real big data applications that may have more diverse benefits.

## Figures and Tables

**Table 1:** Properties of Data Sets.

Name of the Dataset	Objects	Feature	Class
Phoneme	5404	5	2
Kin8nm	8192	8	2
mozilla4	15,545	5	2

**Table 2:** Parameter settings of Spark-MOPSO-Avg algorithm.

Description	Parameters	Value
Particle number	$N_{p}$	50
Maximum iterations	Itermax	30
Archive number	Repository	15
Partition Number	*n*	200
Weight factor	*w*	3
learning parameters	*c*_1_	1.49445
learning parameters	*c*_2_	1.49445

**Table 3:** Wilcoxon test results for the Running time metric, the symbol ✔ means that there is significant difference.

Dataset	Spark-MOPSO-Avg vs. MOPSO-Single	Spark-MOPSO-Avg vs. Spark-MOPSO-Particle	Spark-MOPSO-Avg vs. Spark-MOPSO-labelPartition
phoneme	✔	✔	✔
Kin8nm	✔	✔	✔
Mozilla4	✔	✔	✔

**Table 4:** The Statistical results of accuracy measures in each data set for Spark-MOPSO-Avg, Spark-MOPSO-Particile and MOPSO-Single.

Dataset	Algorithm	Best	Worst	Average	StDev.
phoneme	Spark-MOPSO-Avg	0.7756	0.5973	0.6973	0.0595
	Spark-MOPSO-Particle	0.7296	0.6737	0.6907	0.0197
	MOPSO-Single	0.7285	0.7507	0.6843	0.0282
Kin8nm	Spark-MOPSO-Avg	0.6980	0.5966	0.6317	0.0342
	Spark-MOPSO-Particle	0.6854	0.5871	0.6391	0.0362
	MOPSO-Single	0.6611	0.6118	0.6355	0.0157
Mozilla4	Spark-MOPSO-Avg	0.6480	0.5935	0.6381	0.0165
	Spark-MOPSO-Particle	0.8024	0.6910	0.7468	0.0407
	MOPSO-Single	0.8062	0.6833	0.7347	0.0389

**Table 5:** The Statistical results of accuracy measures in each dataset for Spark-MOPSO-Avg and Spark-MOPSO-label Partition.

Dataset	Algorithm	Best	Worst	Average	StDev.
phoneme	Spark-MOPSO-Avg	0.7756	0.5973	0.6973	0.0595
	Spark-MOPSO-labelPartition	0.7089	0.5719	0.6471	0.0365
Kin8nm	Spark-MOPSO-Avg	0.6980	0.5966	0.6317	0.0342
	Spark-MOPSO-labelPartition	0.6612	0.5870	0.6211	0.0330
Mozilla4	Spark-MOPSO-Avg	0.6480	0.5935	0.6381	0.0165
	Spark-MOPSO-labelPartition	0.6132	0.5930	0.6071	0.0064

**Table 6:** Wilcoxon test results for the accuracy rate metric.

Dataset	Spark-MOPSO-Avg vs. MOPSO-Single	Spark-MOPSO-Avg vs. Spark-MOPSO-Particle	Spark-MOPSO-Avg vs. Spark-MOPSO-labelPartition
phoneme	✘	✘	✘
Kin8nm	✘	✘	✘
Mozilla4	✔	✔	✔

**Table 7:** Detailed table of experimentaldata sets.

Name of the Dataset	Objects	Feature	Class
Dataset1	10,000	2	3
Dataset2	50,000	2	3
Dataset3	100,000	2	3
Dataset4	200,000	3	3

## Data Availability

The article contains the data which are also available from the corre-sponding authors upon reasonable request.
